# An integrated multi-algorithm analysis to decipher senescence and metabolic characteristics in nucleus pulposus cells during disc degeneration

**DOI:** 10.1007/s11357-025-01962-6

**Published:** 2025-11-12

**Authors:** Zhuangyao Liao, Ming Li, Ziyu Chen, Zhi Yao, Yuluan Wu, Zhen Tan, Junyu Qian, Chunyuan Yang, Lin Huang, Lixiang Xue, Deli Wang

**Affiliations:** 1 Department of Bone and Joint Surgery, Peking University Shenzhen Hospital, Shenzhen Peking University-The Hong Kong University of Science and Technology Medical Center, Shenzhen, 518000 Guangdong China; 2 Cancer Center of Peking University Third Hospital, Center of Basic Medical Research, Institute of Medical Innovation and Research, Peking University Third Hospital, Beijing, 100000 China; 3 Department of Orthopedics, Sun Yat-Sen Memorial Hospital, Sun Yat-Sen University, Guangzhou, China

**Keywords:** Senescence, Nucleus pulposus, IVDD, Bioinformatic analyses

## Abstract

Accumulating evidence has suggested that intervertebral disc degeneration (IVDD) serves as a health problem deserving attention from worldwide, owing to the low back pain and even disability it causes. Cellular senescence, an irreversible process of terminal cell cycle arrest, plays an essential role in the degeneration of nucleus pulposus (NP) cells and IVDD with an unknown underlying mechanism. Here, we have explored the senescence signature in IVDD through high dimensional weighted gene co-expression network analysis (hdWGCNA) algorithm in two single cell datasets (PRJCA014236 and GSE244889) to construct a novel signature called NP_Senescence, which accurately identifies the patients with IVDD. The efficacy and accuracy were validated in various external datasets, including two expression array datasets (GSE70362 and GSE34095) and one single cell dataset (GSE230809). Additionally, the accuracy of the specific model was also verified with the senescent cell identification (SenCID) algorithm. IVDD patients with a high NP_Senescence score exhibited the characteristics of compensated activation of hyaluronic acid monomers synthesis and abnormal status of DNA damage repair through single-cell Flux Estimation Analysis (scFEA) algorithm. Furthermore, the NP_Senescence was negatively associated with the level of AMP and Glucose-1-phosphate, while positively associated with the level of β-alanine and UDP-glucuronic acid. In conclusion, we constructed a novel disc-specific scoring model NP_Senescence by comprehensively analyzing the senescence-related genes of IVDD, which can be used for patient early diagnosis and treatment, as well as the identification of metabolic characteristics.

**Supplementary Information:**

The online version contains supplementary material available at https://doi.org/10.1007/s11357-025-01962-6.

## Introduction

Intervertebral disc degeneration (IVDD), one of the leading causes of low back pain, has the characteristics of high morbidity and onset across multiple age groups [1]. The prevalence of IVDD, which may induce various spine-related diseases such as spinal stenosis and disc herniation, has caused a severe societal financial burden nowadays [2]. However, the underlying mechanisms of IVDD remain unclear, leading to the deficiency of treatments, which are limited to symptomatic treatment with analgesic drugs or surgical treatment. Therefore, there is an urgent need for a novel and precise diagnostic predictor for better early intervention.

The intervertebral disc tissue is mainly composed of gelatinous tissue called nucleus pulposus (NP), annulus fibrosus (AF) tissue on both sides, and cartilage endplates covering the upper and lower sides. Among them, the NP tissue is considered to be the essential functional component of the intervertebral disc tissue. The onset of IVDD results from the reduction in functional NP cells and the disruption in matrix metabolism, which is attributed to various factors, including cellular senescence. Cellular senescence is characterized by the status of permanent proliferation arrest and biological dysfunction of cells, which is driven by sustained stimulation [3]. Senescence has been reported as an essential trigger for the degeneration of NP cells. Compared with disc samples from non-herniated patients, IVDD samples exhibited a higher presence of SA-β-gal positive senescent NP cells [4]. Meanwhile, it was observed that the senescence-associated secretory phenotype (SASP) was abnormally activated in degenerated NP cells [5, 6], suggesting the indispensable role of senescence in the process of degeneration. Considering the close relationship between senescence and IVDD, there are several studies focusing on exploring novel targets and corresponding senolytics on NP cells [7, 8]. Silence of KMT2A could suppress the SASP phenotype through enhancing autophagic flux, implying that KMT2A may be an ideal target for anti-senescence therapy [9]. Another study revealed that knockdown of EGR1 could retard the senescence of NP cells by the PINK1-Parkin dependent mitophagy [10]. Additionally, senolytics based on specific targets or pathways have also been reported to potentially play a role in eliminating senescent cells in intervertebral disc degeneration. Curcumin and o-Vanillin can reduce SASP factors and increase matrix synthesis through the Nrf2 and NFkB pathways [11]. Another natural compound, Quercetin, can inhibit the activation of the NF-κB pathway cascades and further stimulation of SASP factor expression both induced by IL-1β in NP cells [12]. However, the limitations of only a single cohort in these studies hindered the development and clinical application of corresponding senolytics.

With the rise of single-cell sequencing technology in recent years, researchers are able to explore IVDD with more precise resolution on multiple cohorts. Previous studies have reported a senescence signature in IVDD mainly based on expression data of microarray [13, 14], whose accuracies were limited by the sample size and chip accuracy. Thus, there is an urgent need for a more accurate and robust predictor or target on NP cell senescence, which is conducive to the early diagnosis and treatment of IVDD. At the same time, it is well known that senescence is closely associated with metabolic regulation, such as in redox balance [15], DNA damage [16, 17], and mitochondrial dysfunction [18], which is difficult to study in depth at the tissue level because of the insufficient sequencing depth. Recent studies developed a variety of single-cell algorithms that can help unearth the pathological mechanisms, such as the cellular metabolism of senescent NP cells [19, 20]. Hence, the assessment of the underlying mechanism in senescent NP cells at a single cell level benefits the early diagnosis and drug development based on senescence.

In the present study, we integrated multiple single-cell datasets from public databases for a comprehensive evaluation of senescence in IVDD, and found the abnormal activation of senescence in degenerated NP cells. Delving deeper, we utilized multiple single-cell advanced algorithms and constructed a novel signature called NP_Senescence, which was correlated with the activation of metabolic pathways associated with senescence and the concentration of metabolites. Meanwhile, we further compared and validated the signature with the previously reported senescence assessment algorithms, and verified its predictive efficiency of diagnosis in external single-cell dataset and conventional assay datasets. Our findings indicate that the NP_Senescence is an efficient and robust signature for early diagnosis of IVDD, and the genes involved in the signature may be the potential senescence-related targets.

## Materials and methods

### The acquisition and preprocessing of public data about IVDD

The single cell data of IVDD tissue was downloaded from the PRJCA014236, GSE244889, and GSE230809 datasets. The microarray data with clinical information of human IVDD tissue was obtained from the GSE70362 and GSE34095 datasets. The GSE70362, GSE34095, GSE244889, and GSE230809 datasets were accessed and downloaded from the Gene Expression Omnibus (GEO) database, while the PRJCA014236 dataset was acquired from the Genome Sequence Archive (GSA) database.

For the single-cell data, we performed quality control, batch effect removal, dimensionality reduction, and cell clustering with the R package Seurat [21] and harmony [22]. According to markers reported in a previous study [23], we annotated the cell types of the intervertebral disc microenvironment. After extracting NP cells, we performed a function called AddModuleScore to evaluate the activation of cellular senescence. Subsequently, we applied high-dimensional weighted gene co-expression network analysis (hdWGCNA) to explore the correlation between the senescence score and module [20]. We then adopted single-cell Flux Estimation Analysis (scFEA) to estimate the concentration of metabolites and the activation of the metabolic process [19]. Afterwards, we implemented a senescent cell identification (SenCID) algorithm, an algorithm for senescence assessment, to identify the characteristics and degree of senescent NP cells [24] (Supplemental Fig. 1).

For the microarray data with probe annotation, we performed quality control and normalization before analyses.

The WP_Ferroptosis and KEGG_Ferroptosis gene lists were downloaded from the MSigDB database GSEA (https://www.gsea-msigdb.org/gsea) hallmark gene sets.

The version numbers for all software packages are listed in Supplemental Table 1.

### Gene Ontology (GO) and Kyoto Encyclopedia of Genes and Genomes (KEGG) functional enrichment analysis

Function enrichment analysis was performed with both GO and KEGG categories using R package clusterProfiler [25] on hub genes obtained from the module positively correlated with high Senmayo score. The p-values were adjusted using “BH” (Benjamini–Hochberg) correction. GO terms, including biological process (BP), cellular component (CC), and molecular function (MF), were ranked by q value. Enrichments for KEGG were also sorted by gene counts and p value. Both enrichments were considered significant with the cutoff of 0.05.

### High dimensional weighted gene co-expression network analysis

Gene co-expression network analysis was performed to explore the gene modules correlated with the Senmayo score in NP cells using the hdWGCNA package. Followed parameters were set to conduct hdWGCNA analysis: k = 25, assay = ‘RNA’, max_shared = 10, min_cells = 50, reduction = ‘harmony’, slot = ‘data’. The ConstructNetwork function was applied to construct the co-expression network with the soft threshold automatically detected of Scale-free topology model fit greater than 0.8. Module detection was performed with the minimal module size set to 50 genes, and the highly correlated modules were merged with the cutoff tree height of 0.2. For each module, the first principal component, called eigengene, which reflected the specific expression pattern, was calculated, and the intra-modular connectivity, which was representative of the correlation with the module eigengene, was estimated for each gene with the SignedKME algorithm. Modules with positive correlation to a high Senmayo score were selected for subsequent analyses.

### Single-cell flux estimation analysis

The scFEA software was implemented through Python to predict the metabolic flux and metabolites' abundance changes of each NP cell based on flux balance constraints and the novel graph neural network-based architecture algorithm. The original expression matrix of NP cells, and three tables including the information of modules and corresponding genes, metabolite names and corresponding ID numbers, and the relationship matrix between metabolites and metabolic modules were input as required files. Tables for the last three corresponding species are provided on the software website. After routine normalization and software analysis, the output tables including metabolic fluxes and metabolites for each cell and module were obtained. Differences in the metabolites and modules of different NP cells grouped by the Senmayo score were then calculated and visualized.

### Single-cell senescence identification analysis

The SenCID analysis was conducted based on Python software to identify the characteristic and classification of NP cells grouped by our novel signature NP_Senescence. The original expression matrix of NP cells and the reference files officially provided about six major senescence identities, which reflect different senescence baselines, stemness, gene functions, and responses to senolytics, were input. The cells in the expression matrix were automatically recognized as one of the six identities and assigned a corresponding senescence score. The difference in SID scores, which was considered a reference index of NP_Senescence prediction accuracy, between groups divided by the novel signature was then calculated.

### Development of a novel signature NP_Senescence

The common genes were utilized to construct a novel signature called NP_Senescence, which is specifically suitable for predicting the senescent degree of nucleus pulposus cells. For scRNA-seq data, we calculated NP_Senescence through the Seurat function “AddModuleScore”, which averaged the expression of the common genes in each single cell. For the microassay data, we evaluated the diagnostic performance of genes included in the signature. The performance was visualized by receiver operating characteristic (ROC) curve and calculated the area under the ROC curve (AUC) in the external validation set.

### Statistical analysis

Correlations between variables were estimated with Spearman correlation analysis through the R package ggpubr. On the premise of continuous variables, the t test or Wilcoxon test was used to calculate differences between two groups, while Kruskal–Wallis tests were performed for more than two groups. All statistical analyses were carried out with R software (v.4.0.2). Two-sided *P* values < 0.05 were considered statistically significant.

## Results

### Nucleus pulposus cell senescence is abnormally activated in intervertebral disc degeneration

**Fig. 1:**
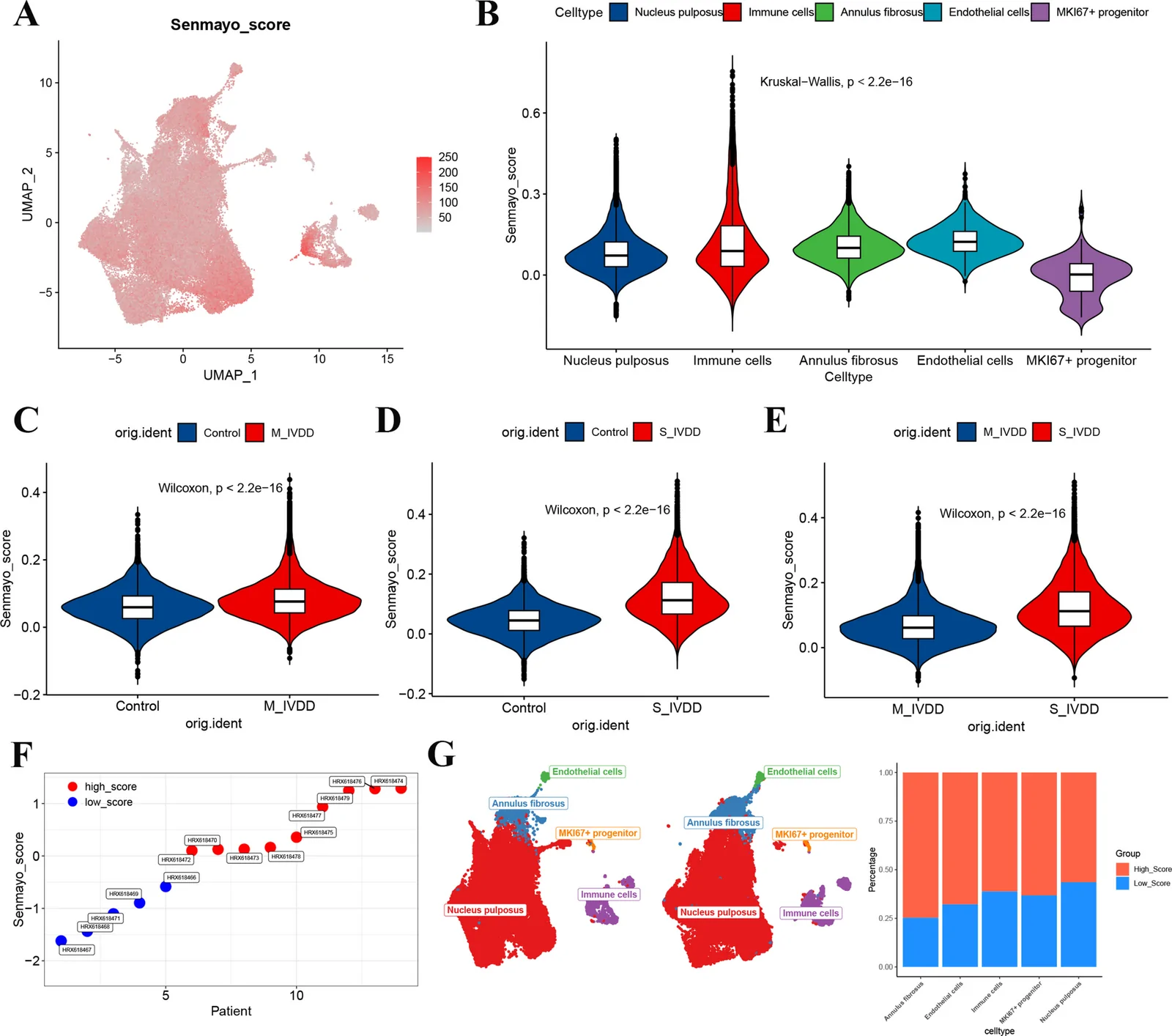
The senescence landscape of NP cells in PRJCA014236 dataset. **A** UMAP plot of Senmayo score in PRJCA014236 dataset. **B** Violin plot of Senmayo score in different cell types. **C** Violin plot of Senmayo score in control NP cells and moderately degenerated NP cells. **D** Violin plot of Senmayo score in control NP cells and severely degenerated NP cells. **E** Violin plot of Senmayo score in moderately and severely degenerated NP cells. **F** Senmayo score of different samples. **G** The UMAP plot and stacking plot revealing the characteristic and proportion of high (left) and low (right) Senmayo score group

To investigate the activation state of cell senescence in IVDD, we firstly extracted NP cells from the public dataset PRJCA014236 and performed geneset scoring with the universal senescence geneset Senmayo. Interestingly, among the five cell types, NP cells did not exhibit the highest senescence score at baseline (Fig. 1A–B), which indicated that a high baseline NP cell senescence score is not a specific characteristic of intervertebral disc degeneration. We further compared the senescence score of NP cells in different grades of IVDD and found the Senmayo score was significantly elevated as the grade of NP cell degeneration increases (Fig. 1C–E). At the same time, we integrated the senescence score in different samples and separated the samples into a high score group and a low score group, with 9 cases in the former and 5 cases in the latter (Fig. 1F). Interestingly, higher scores were mainly distributed in samples with high levels of degeneration. The UMAP plot revealed the characteristics of cells in the intervertebral disc microenvironment in patients with high scores and low scores (Fig. 1G). The stacking plots exhibited that patients in the high score group have higher infiltration levels of most cell types, including Annulus fibrosus, Endothelial cells, and immune cells (Fig. 1G), except for nucleus pulposus cells. Considering NP cells are the initiating cells and functional cells during the degeneration of the intervertebral disc, we extracted NP cells for subsequent analyses.

### Identification of hub genes and characteristics of high senescence score

**Fig. 2:**
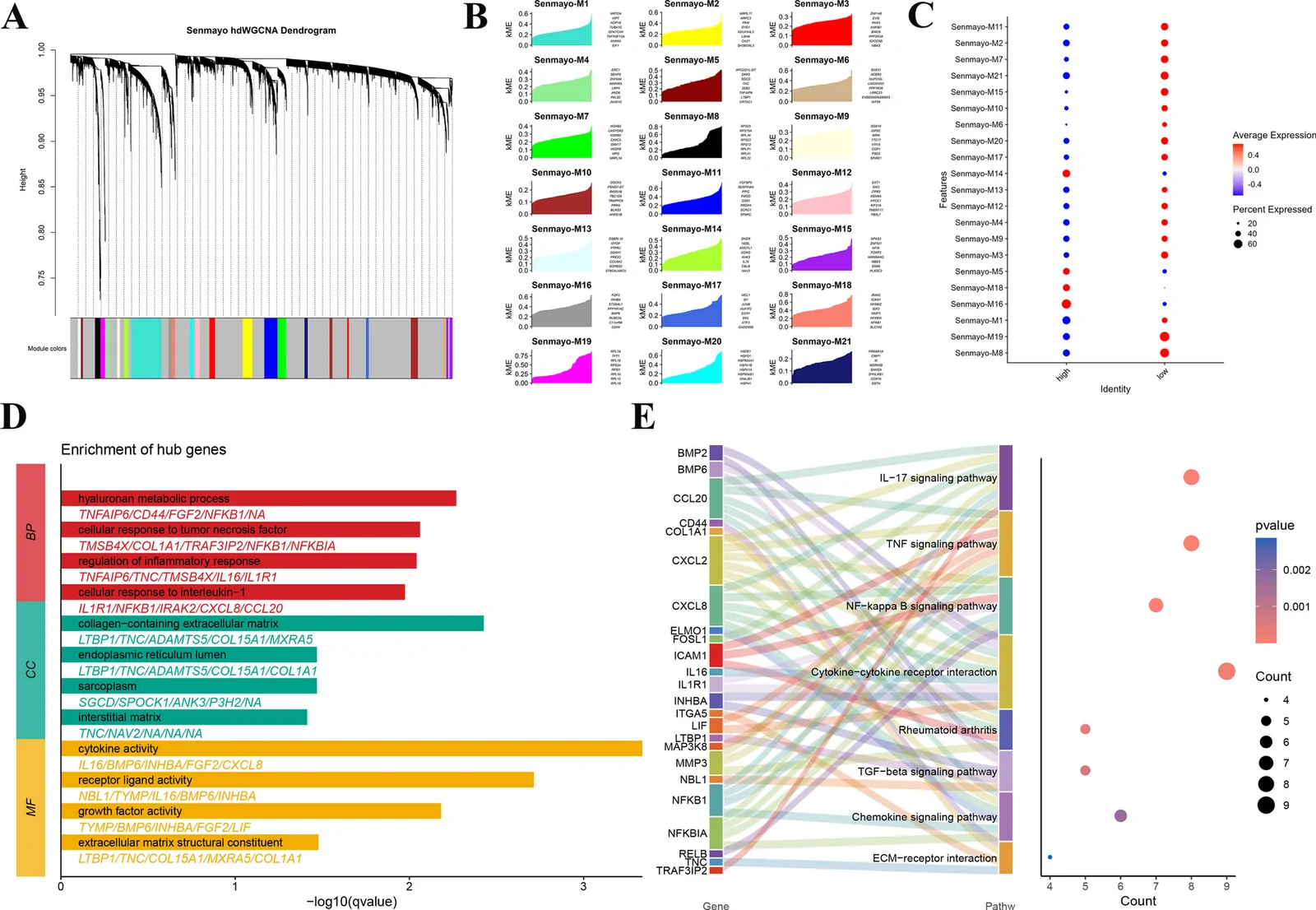
HdWGCNA analysis of NP cells with high Senmayo score in PRJCA014236 dataset. **A** Co-expression network diagram of NP cell with high Senmayo score. **B** Modules and associated top hub genes displayed according to the hdWGCNA pipeline. **C** Correlation diagram between modules and Senmayo score. **D** Bar diagram of GO enrichment of hub genes, including biological process (BP), cellular component (CC) and molecular function (MF). **E** Alluvial diagram and bubble diagram of KEGG enrichment analysis of hub genes

To explore the underlying hub genes in high senescence NP cells, we conducted hdWGCNA with the Senmayo score. We firstly selected the optimal soft threshold to make excellent consistency between the network’s topology and the biological information. Subsequently, we constructed the hierarchical co-expression network visualized by the dendrogram through measuring gene expression similarity, calculating the topological overlap matrix, and performing hierarchical clustering (Fig. 2A). Next, we obtained representative genes from each module, and the correlation of each gene within modules was exhibited by the connectivity plot (Fig. 2B). Meanwhile, we calculated the correlation between modules and senescence scores and found that Module-5, 14, 16, and 18 were significantly correlated with high senescence scores (Fig. 2C). We then extracted the top 25 central genes in the above modules and applied function enrichment analyses in GO and KEGG databases. In GO biological process pathways, the classical pathways involving inflammation and matrix metabolism, such as cellular responses to tumor necrosis factor (TNF) and interleukin-1, hyaluronan metabolic process was highly enriched. Meanwhile, the collagen-containing extracellular matrix (ECM), endoplasmic reticulum lumen, and similar cellular components participating in the degeneration of NP cells were also significantly enriched [26, 27]. In the category of molecular function, the cytokine activity [28] and extracellular matrix structural constituent involved in the senescence of NP cells were also activated (Fig. 2D). Moreover, the hub genes also showed prominent associations with pathways involved in senescence and ECM metabolism of NP cells, such as TNF signaling pathway [5], transforming growth factor-β (TGF-β) signaling pathway [29] and ECM-receptor interaction pathway [30] (Fig. 2E), which verified the correlation between the hub genes and senescence of NP cells.

### Validation of the abnormal activation of NP cell's senescence in IVDD

**Fig. 3:**
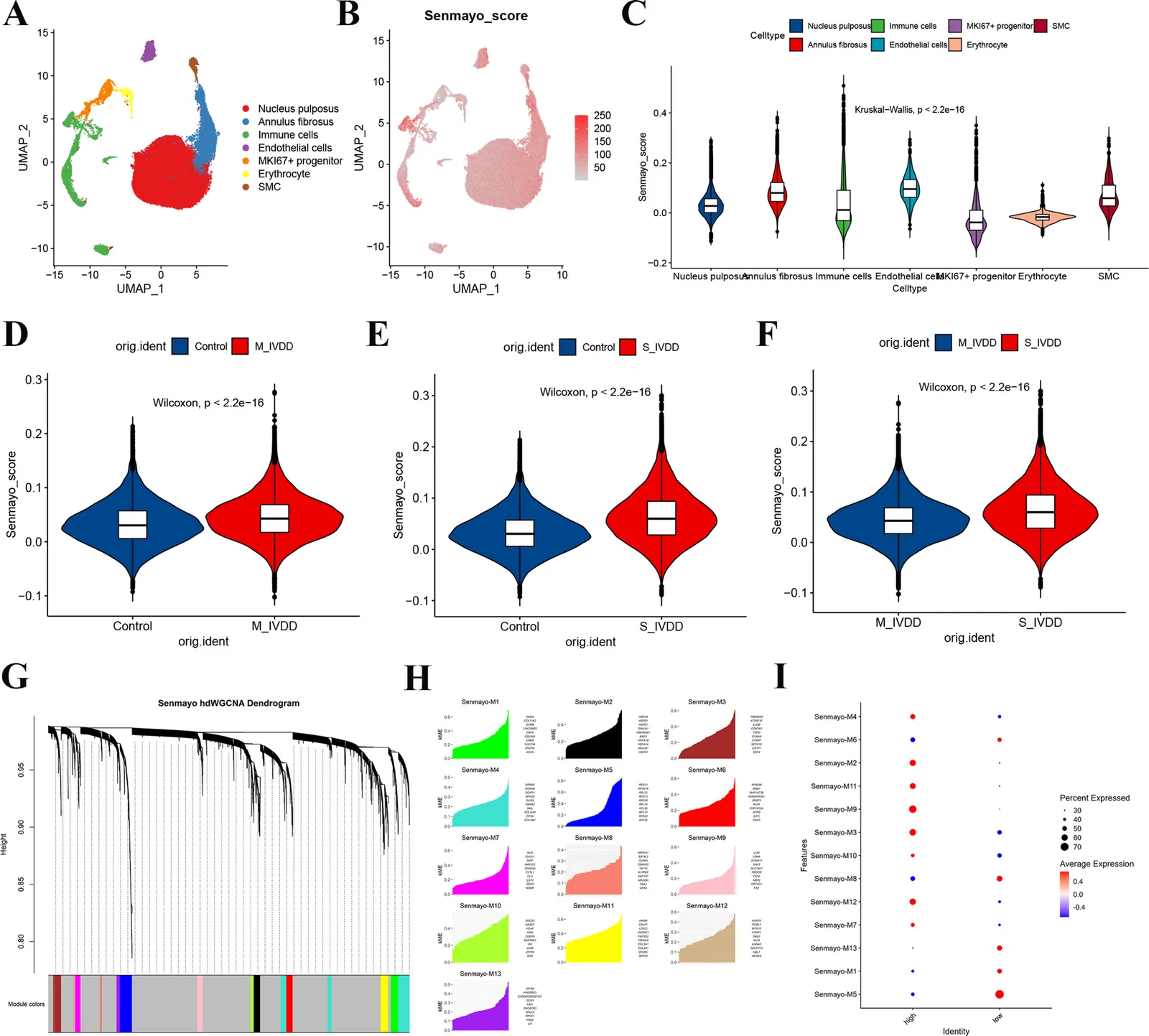
The senescence landscape of NP cells in GSE244889 dataset. **A** UMAP plot of cell types in GSE244889 dataset. **B** UMAP plot of Senmayo score in GSE244889 dataset. **C** Violin plot of Senmayo score in different cell types. **D** Violin plot of Senmayo score in control NP cells and moderately degenerated NP cells. **E** Violin plot of Senmayo score in control NP cells and severely degenerated NP cells. **F** Violin plot of Senmayo score in moderately and severely degenerated NP cells. **G** Co-expression network diagram of NP cell with high Senmayo score. **H** Modules and associated top hub genes displayed according to the hdWGCNA pipeline. **I** Correlation diagram of modules and Senmayo score

**Fig. 4:**
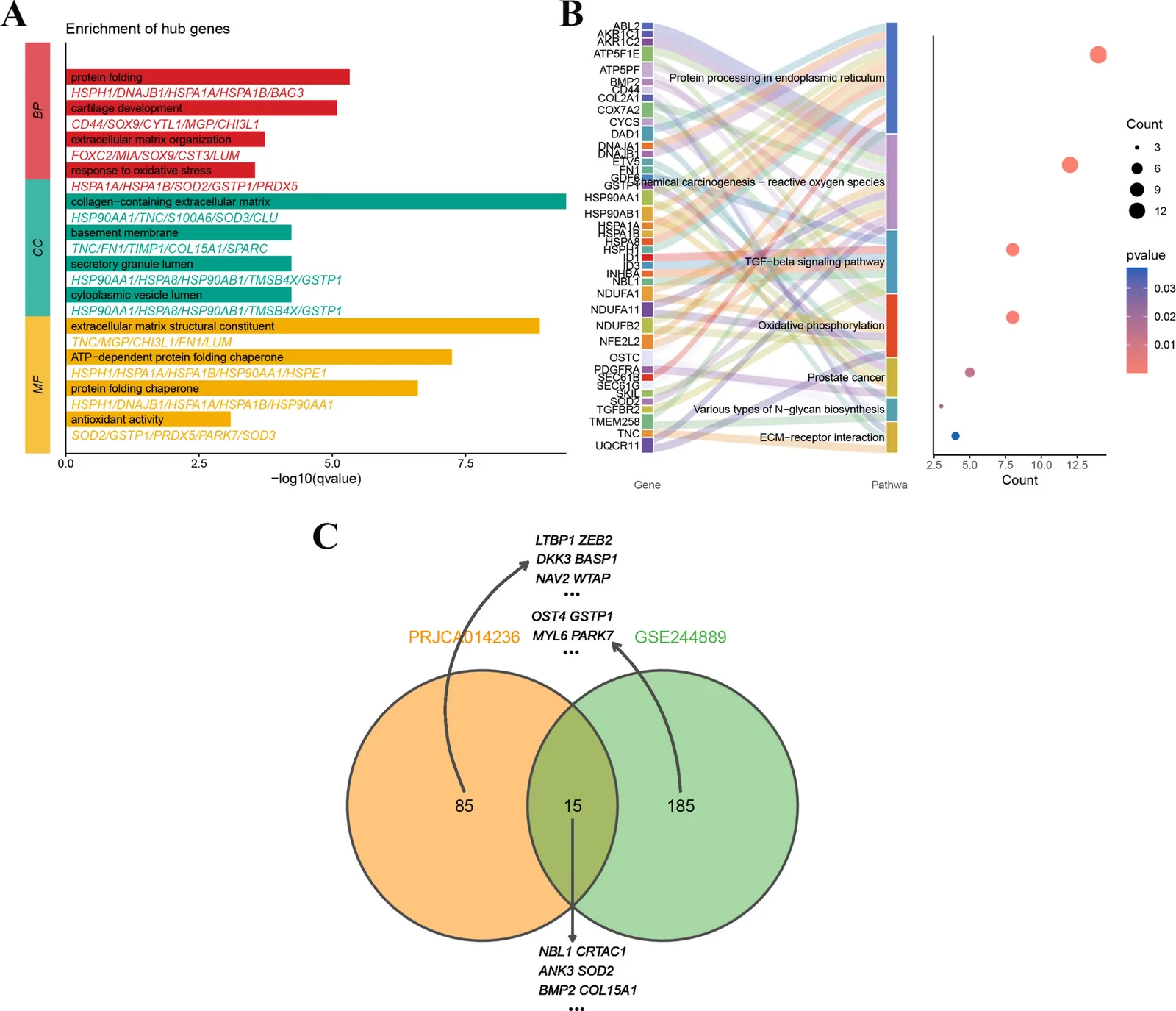
Biological characteristics of hub genes in the GSE244889 dataset. **A** Bar diagram of GO enrichment analysis of hub genes. **B** Alluvial diagram and bubble diagram of KEGG enrichment analysis of hub genes. **C** Venn diagram of common hub genes (genes indicated by the arrow only for exhibition)

To validate the robustness of the above outcomes, we collected another single cell dataset, GSE244889. After quality control, dimensionality reduction, and clustering, we also annotated cell types with specific cell markers (Fig. 3A–B). Consistently, there was no high baseline NP cell senescence score based on the Senmayo geneset in this dataset (Fig. 3C), and the activated senescence state was also observed in high-grade degeneration samples (Fig. 3D–F). Subsequently, we conducted hdWGCNA analysis in this dataset and found Module-2, 3, 4, 7, 8, 9, 10, and 12 were positively correlated with senescence scores (Fig. 3G–I). Function enrichment analysis revealed the extracted hub genes were similarly enriched in the pathway regarding specific characteristics of NP cells, such as cartilage development, extracellular matrix organization, and structural constituent (Fig. 4A). Meanwhile, the above hub genes were also associated with pathways involved in the activation of senescence in the nucleus pulposus, for example, secretory granule lumen [31] and ATP-dependent protein folding chaperone [32]. KEGG enrichment analysis gave similar results that TGF-β signaling pathway, Longevity regulating pathway, and ECM-receptor interaction were obviously enriched (Fig. 4B), which validated the effectiveness of the above hub genes. We then intersected the hub genes in two datasets and obtained 15 genes including NBL1, CRTAC1, ANK3, SOD2, BMP2, COL15A1, TNC, TMSB4X, PAPSS2, IL1R1, GLIS3, CD44, INHBA, GPRC5A, and IER3 (Fig. 4C and Supplemental Table 3).

### Construction of the novel signature called NP_Senescence

**Fig. 5:**
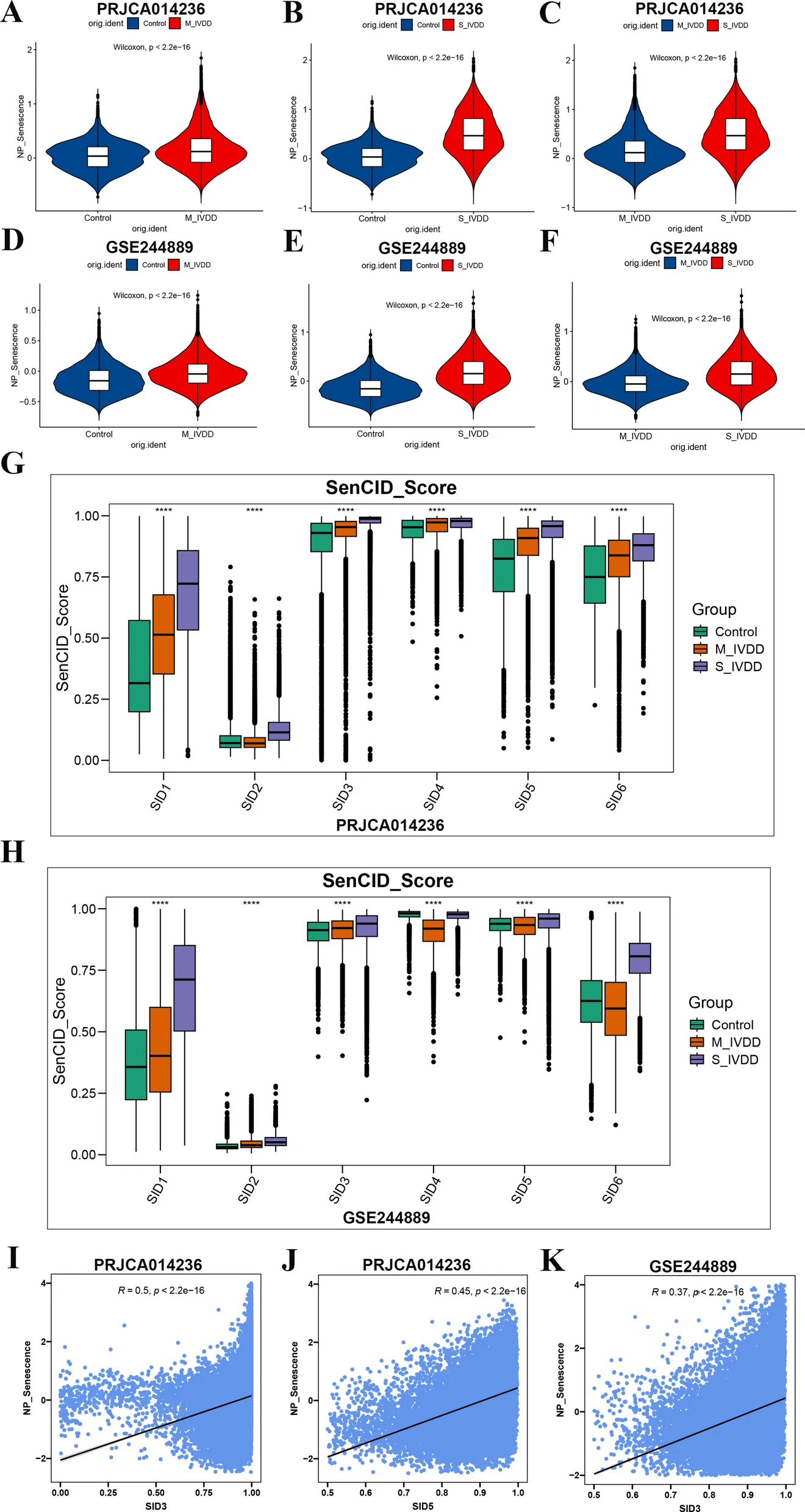
Quantification system based on the novel signature NP_Senescence. **A**–**C** Violin plot of NP_Senescence in PRJCA014236 dataset. **D**–**F** Violin plot of NP_Senescence in GSE244889 dataset. **G** Boxplot of SenCID score in PRJCA014236 dataset. **H** Boxplot of SenCID score in GSE244889 dataset. **I** Correlation diagram of NP_Senescence and SID3 score in PRJCA014236 dataset. **J** Correlation diagram of NP_Senescence and SID5 score in PRJCA014236 dataset. **K** Correlation diagram of NP_Senescence and SID3 score in GSE244889 dataset

Considering the classic Senmayo geneset containing too many genes, which limited it applicability, we constructed a novel signature for nucleus pulposus called NP_Senescence containing above 15 genes. We validated this signature in above two single cell datasets, and found the significant increase of the score in the high Pfirrmann grade samples. Compared with the Senmayo score, the difference in NP_Senescence score between different grades was more obviously, not only in PRJCA014236 (Fig. 5A–C), but also in GSE244889 (Fig. 5D–F). For verifying the effectiveness of the score, we applied SenCID analysis, a machine learning program which could accurately and automatically identify six major senescence identities (SID1-6) and calculate corresponding identity scores (SenCID scores) spanning 30 cell types, including chondrocytes. In PRJCA014236 dataset, the SenCID scores across five identities exhibited an overall increase trend with degeneration levels, except SID2 (Fig. 5G). The vast majority of NP cells were identified as the SID3 category, which was reported to show enrichment for mitochondria and redox reactions. The remaining one thirds of NP cells were recommended as the SID5 category with the specific characteristic of high activity about BCL2 family genes, which participated in the senescence-related NP degeneration [33]. Moreover, consistent with above results, NP cells in GSE244889 dataset were also divided into SID3 pattern, which also showed an increasing trend as the grade of degradation increases (Fig. 5H). Interestingly, it is reported that among the 30 cell types, chondrocytes in osteoarthritis were also detected as SID3 cells, which, to a certain extent, explained the homologous between chondrocytes and NP cells. We further calculated the correlation between NP_Senescence and SenCID score of corresponding categories, and the results showed a significant correlation in both two datasets (Fig. 5I–K), which indicated the effectiveness of our novel signature NP_Senencence.

### Identification of metabolism-specific phenotypes in senescent NP cells

**Fig. 6:**
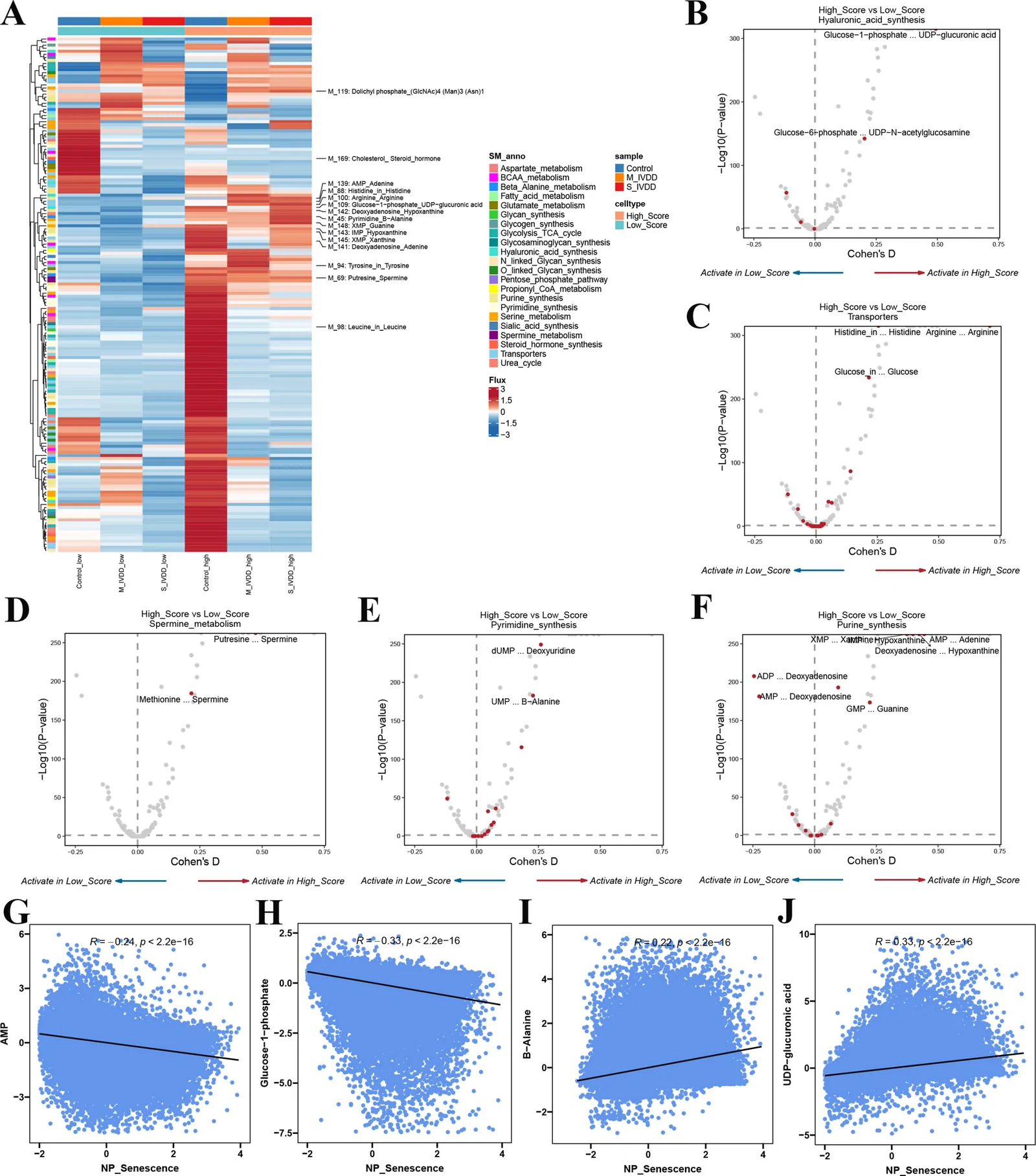
Metabolic characteristic of NP cells based on scFEA algorithm in PRJCA014236 dataset. **A** Heatmap of specific metabolic landscape of NP cells grouped by NP_Senescence. **B**–**F** Volcano diagram of metabolic pathways hyaluronic acid synthesis, transporters, spermine metabolism, pyrimidine synthesis and purine synthesis. **G**–**J** Correlation diagrams of NP_Senescence with AMP, glucose-6-phosphate, β-aianine and UDP-glucuronic acid

**Fig. 7:**
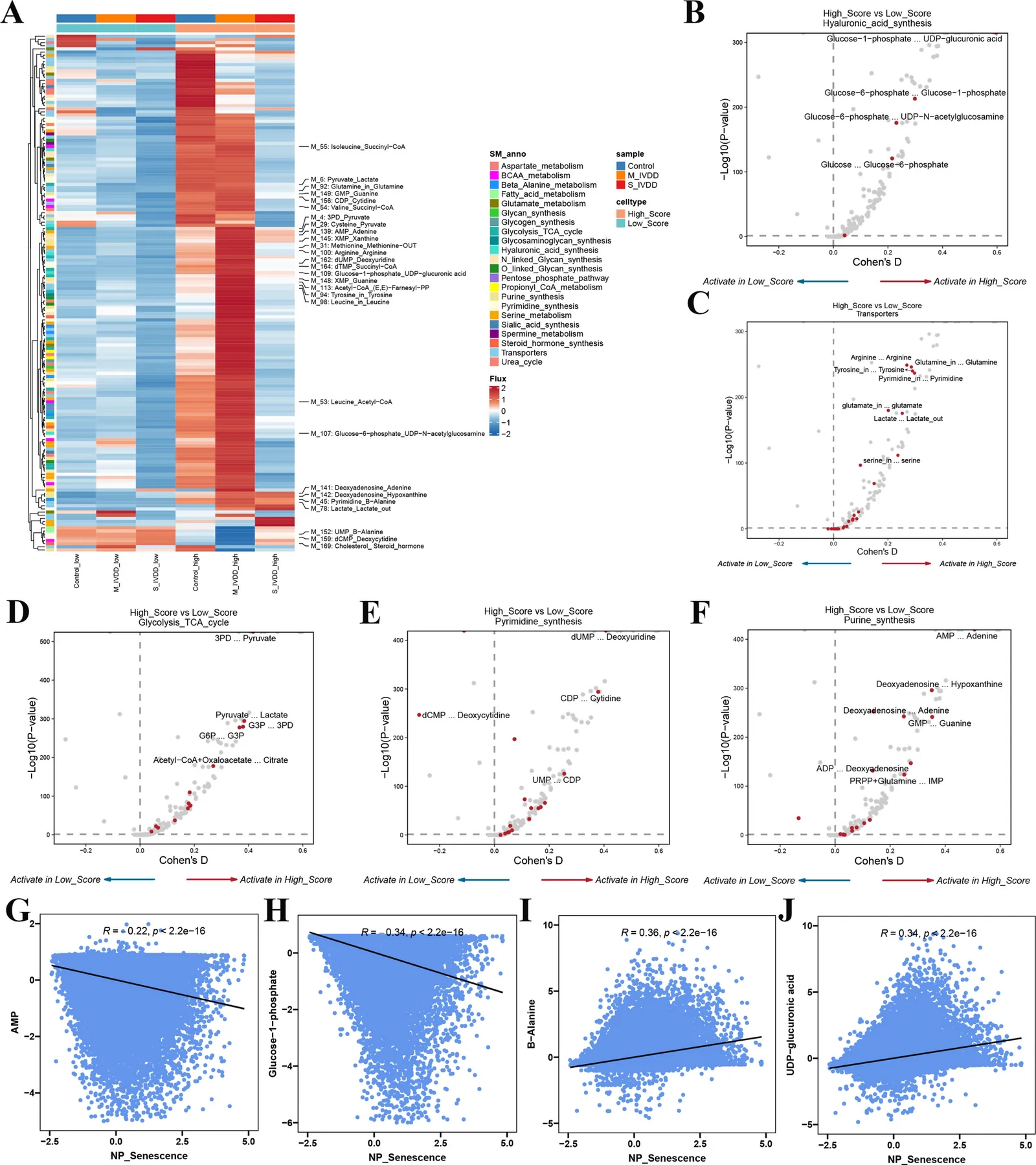
Metabolic characteristic of NP cells based on scFEA algorithm in GSE244889 dataset. **A** Heatmap of specific metabolic landscape of NP cells grouped by NP_Senescence. **B**–**F** Volcano diagram of metabolic pathways hyaluronic acid synthesis, transporters, glycolysis TCA cycle, pyrimidine synthesis and purine synthesis. **G**–**J** Correlation diagrams of NP_Senescence with AMP, glucose-6-phosphate, β-aianine and UDP-glucuronic acid

Cell senescence is closely related to metabolic regulation, which can shape the specific senescent phenotype. The imbalance in the redox system, proteostasis, and organelle homeostasis, as well as changes in related metabolites, are involved in the regulation of cell senescence. Therefore, we adopted scFEA, a single-cell level flux estimation algorithm based on a multi-layer neural network, to calculate the underlying metabolites in the process of NP cell senescence for exploring the specific metabolic pattern. Based on the NP_Senescence score, we calculated the metabolic flux of the samples and groups. From an overall perspective, in the PRJCA014236 dataset, metabolic pathways including hyaluronic acid synthesis, transporters, spermine metabolism, and purine synthesis were significantly activated in the high NP_Senescence group (Fig. 6A). In the process of hyaluronic acid synthesis, the synthesis of two monomers, UDP-N-acetylglucosamine and UDP-glucuronic acid, required for hyaluronic acid was obviously activated (Fig. 6B). However, this is inconsistent with our common knowledge that extracellular matrix anabolism is downregulated in senescent cells, which indicated that negative feedback may increase monomer synthesis in senescent cells, but hyaluronic acid synthesis and transport may be blocked to some extent during senescence. Moreover, the transport of multiple metabolites, like tyrosine and arginine, and spermine metabolism were also upregulated (Fig. 6C–D). Cells are driven into the senescent state by a series of factors, including telomere shortening and DNA damage [34]. Increased synthesis of β-alanine and deoxycytidine in pyrimidine metabolism was observed in senescent cells (Fig. 6E), which suggested that abnormal DNA damage repair may be in an active state. At the same time, in purine synthesis, there appeared to be an increased demand for various purines in high NP_Senescence score cells, compared with the low score senescent cells. However, the synthesis of deoxyadenosine, whose derivatives have been reported to have anti-senescence effects [35], was significantly inhibited (Fig. 6F). Afterwards, correlation analysis between metabolite concentrations and NP_Senescence revealed that adenosine monophosphate (AMP) and glucose-1-phosphate were negatively correlated with NP_Senescence (Fig. 6G–H), while β-alanine and UDP-glucuronic acid were positively correlated (Fig. 6I–J). In the GSE244889 dataset, the main activated metabolic pathways included hyaluronic acid synthesis, transporters, glycolysis, TCA cycle, and purine synthesis, while pyrimidine synthesis was partially inhibited (Fig. 7A). Similar to the results of the PRJCA014236 dataset, the synthesis of UDP-N-acetylglucosamine and UDP-glucuronic acid was enhanced, as well as glucose-6-phosphate and glucose-1-phosphate, in the process of hyaluronic acid synthesis (Fig. 7B). The transport of multiple metabolites, glycolysis, TCA cycle, and purine synthesis were observed to be abnormally active, while the process of pyrimidine synthesis exhibits dual effects of both activation and inhibition (Fig. 7C–F). Meanwhile, the concentration of AMP and glucose-1-phosphate also showed a negative correlation with NP_Senescence, while the level of β-alanine and UDP-glucuronic acid elevated as the novel signature increased (Fig. 7G–J). On the whole, the senescent NP cells recognized by our novel score may be blocked during hyaluronic acid synthesis and transport and exhibit the characteristic of extraordinarily active DNA mismatch repair.

### External validation on diagnostic efficacy of NP_Senescence

**Fig. 8:**
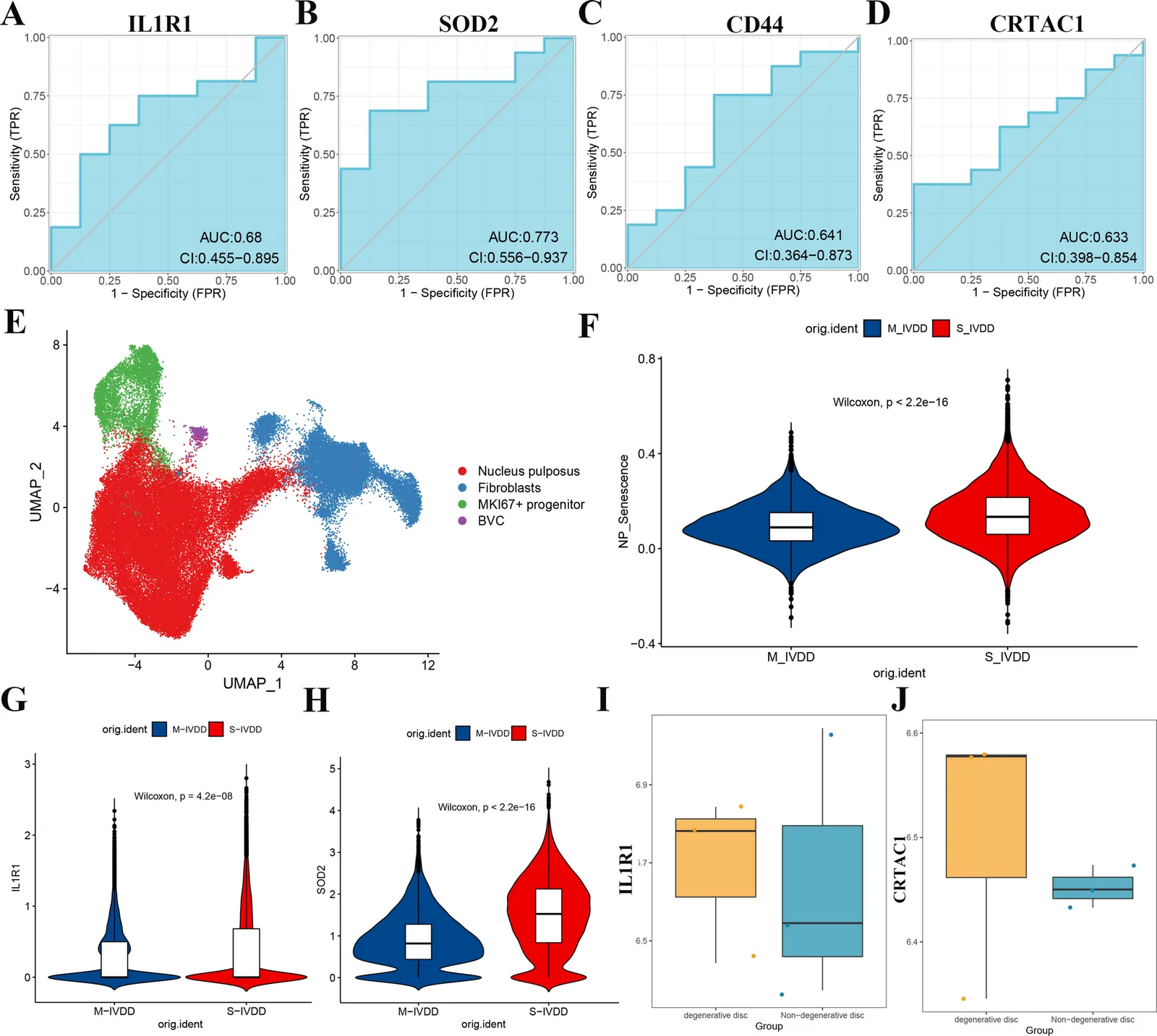
Validation of the NP_Senescence in external validation set. **A**–**D** ROC curves of the marker genes IL1R1, SOD2, CD44 and CRTAC1 in GSE70362 datasets. **E** UMAP plot of cell types in GSE230809 dataset. **F** Violin plot of NP_Senescence in GSE230809 dataset. **G**–**H** Violin plots of IL1R1 and SOD2 expressions in GSE230809 dataset. **I**–**J** Box plots of IL1R1 and SOD2 expressions in GSE34095 dataset

For validating the robustness of the novel signature, we collected the other datasets about IVDD, such as GSE70362, GSE230809, and GSE34095 datasets, to validate the diagnostic efficacy. We found four markers in the signature, namely IL1R1, SOD2, CD44, and CRTAC1, also had a higher predictive accuracy of diagnosis on the external validation set GSE70362, respectively the AUC values of 0.68, 0.773, 0.641, and 0.633 (Fig. 8A–D). On the other hand, in the external single-cell dataset, GSE230809, we also extracted NP cells after conventional dimensionality reduction, clustering, and annotation (Fig. 8E). The specific signature was still observed excellent predictive efficacy, with elevated senescence scores in the high-grade degeneration group (Fig. 8F). Meanwhile, the expression of the markers found above, like IL1R1 and SOD2, was also significantly increased in the severely degenerated group (Fig. 8G–H), which verified their potential to be diagnostic markers. We further validated the effectiveness of our senescence evaluating system in another dataset GSE34095, which only included three pairs of degenerated intervertebral disc and non-degenerated intervertebral disc samples. Due to the limited patient number, the results indicated the trend of increased expression in markers IL1R1 and CRTAC1 regardless of insignificant statistical difference (Fig. 8I–J). Together, these findings indicate that NP_Senescence is a potential and stable predictor of the diagnosis in IVDD.

## Discussion

**Fig. 9:**
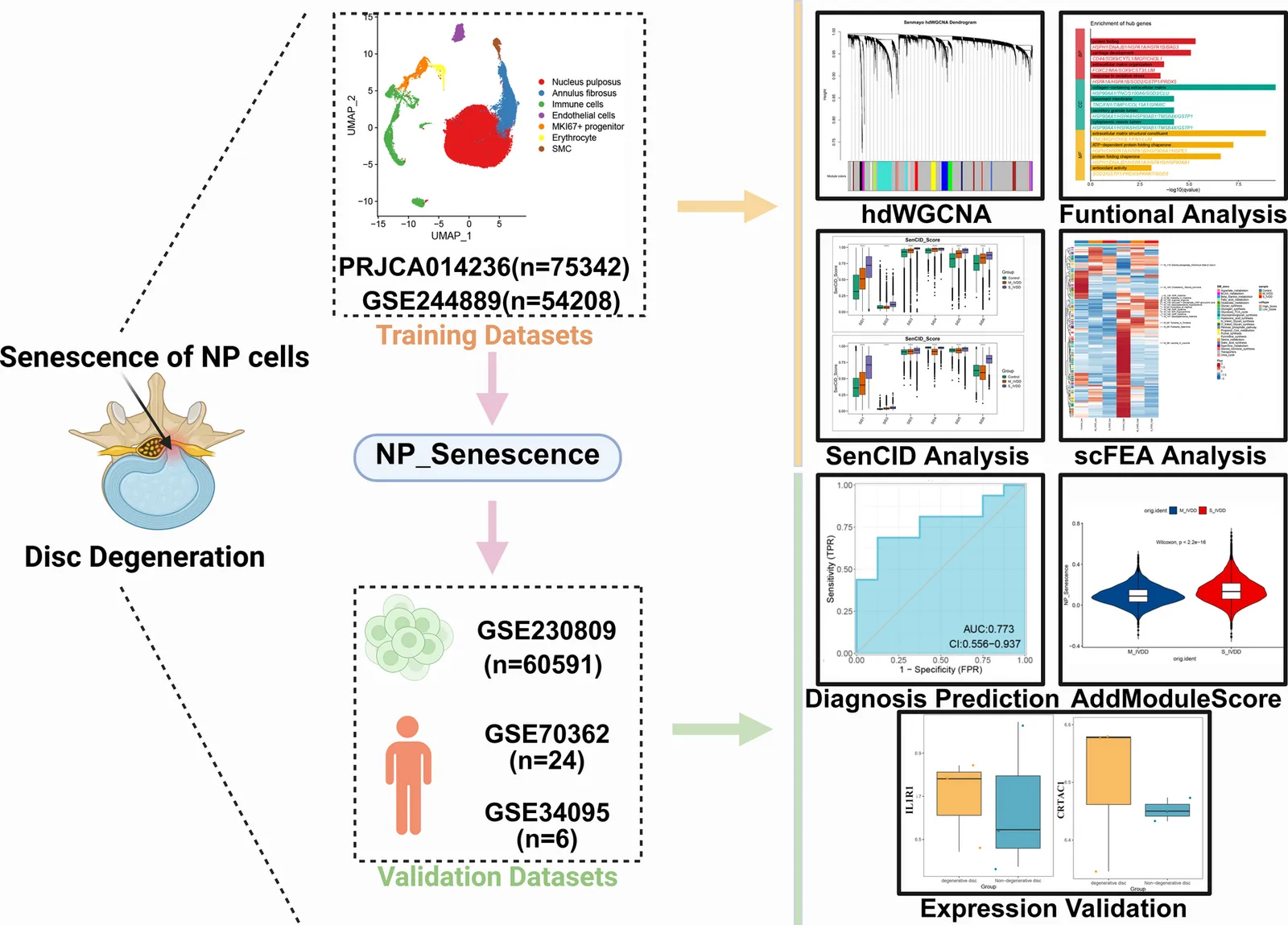
Graphical abstract

In this current study, we analysed the senescence characteristic comprehensively in IVDD by utilizing multiple single cell datasets (PRJCA014236 and GSE244889), and based on various advanced algorithms like hdWGCNA, scFEA, and SenCID to construct a novel signature NP_Senescence, which was further validated in external datasets, including expression arrays datasets (GSE70362 and GSE34095) and another single cell dataset (GSE230809), as shown in Fig. 9.

Senescence, the major cell fate process that every cell faces, is still less understood owing to the complexity and heterogeneity. In the field of IVDD, research on cellular senescence in the NP cells is still in full swing. Due to the specific microenvironment of the intervertebral disc, including hypoxia and mechanical load, DNA damage may occur in the NP cells, which stimulates the accumulation of senescent cells and activation of the SASP phenotype [36]. Cytokines and components secreted by senescent NP cells further impair matrix metabolic homeostasis through fibrosis and structural destruction [37]. Consistent results were observed in the current study. In both PRJCA014236 and GSE244889 single-cell datasets, the significant increase in the senescence score of NP cells with the degeneration grade suggested that senescence is inseparable from the progressive deterioration of the intervertebral disc. On the other hand, heterogeneous SASP is mainly composed of inflammatory factors, chemokines, matrix metalloproteinases, and other secretory components [38], and senescent cells will selectively release different secreted factors under specific conditions. Coincidentally, the enrichment of hub genes obtained by hdWGCNA exhibited that related pathways, such as cellular response to tumor necrosis factor, regulation of inflammatory response, cytokine activity, and growth factor activity, were tightly correlated with SASP, indicating the close relationship between senescence and IVDD.

Accumulating research suggests that metabolic regulation shapes the specific phenotype of senescent cells. The ratio of NAD +/NADH in the redox system, closely correlated with metabolic regulation, involves the evolution of senescence through inducing oxidative stress. An enantiomer of the metabolite 2-hydroxyglutarate called L-2-Hydroxyglutarate, considered a reductive metabolite, can regulate the ratio of NAD +/NADH and reduce reductive stress by inhibiting glycolysis [39]. Another study reported that the disruption of glucose metabolism resulted in an increased ratio of NAD +/NADH, which could be reversed by the extra conversion of lactic acid to pyruvate [40]. Another regulator of redox balance involved in metabolism is the glucose metabolism pathway. NADPH, whose reduced form acts as an antioxidant in oxidative systems, is produced during the conversion of glucose to ribose 5-phosphate and glucose-6-phosphate to 6-glucolactone [41]. Moreover, the abnormally elevated ROS level in senescent cells also requires enough reductive NADPH to eliminate [42]. Interestingly, we found that the reaction of UDP-glucuronic acid synthesized by glucose-6-phosphate was enhanced in the high NP_Senescence score cells, which suggested insufficient raw materials glucose-6-phosphate for NADPH synthesis, and further accumulation of oxidative stress. Otherwise, glucose-1-phosphate was negatively correlated with NP_Senescence, while UDP-glucuronic acid was positively correlated, which also validated the depletion of glucose-6-phosphate because of the active synthesis of UDP-glucuronic acid. Additionally, research has indicated that hyaluronic acid could delay cellular senescence through multiple mechanisms, including regulating CD44 isoform switch in human amniotic epithelial stem cell [43] and stimulating cell proliferation of fibroblasts and keratinocytes [44]. Meanwhile, hyaluronic acid synthesis [45] holds anti-aging properties in keratinocytes and fibroblast cells. We, therefore, proposed the hypothesis that the apparent activation of hyaluronic acid monomer synthesis, as evidenced by our data, is likely mediated by a compensatory negative feedback loop in response to a blockade in hyaluronic acid synthesis and transport. Furthermore, senescent cells typically exhibit reduced DNA repair signals [46]. However, the results in this study showed the characteristic of highly activated purine and pyrimidine synthesis in the high NP_Senescence group, which may suggest high DNA repair capacity. It is well known that high DNA repair capacity can retard the process of cellular senescence [47, 48]. Therefore, we conjectured that extraordinarily active DNA repair in senescent NP cells was stimulated by the DNA damage signals that were constantly identified but could not be repaired. Together, these results in both datasets also prove the effectiveness of our signature to some extent. However, the underlying roles of AMP, β-Alanine, and the pathways of purine and pyrimidine synthesis partially activated in NP cells still need further exploration.

Studies performed over the past few years developed related signatures for IVDD, such as ferroptosis-related [49] and apoptosis-related signature [50]. However, several shortcomings remain unresolved in their research, including the insufficient sample size, shallow analysis depth, and lack of external validation datasets. Our analyses demonstrated that NP_Senescence is an ideal evaluation index for predicting the senescence degree of NP cells in IVDD. One of the hub genes involved in the signature, IL1R1, has been reported as the key gene in IVDD for more than one study [51, 52], but its senescence-related role in IVDD still remains unknown. SOD2, as one member of the superoxide dismutase family, can mediate redox homeostasis through the oxidative stress pathway. Nevertheless, high expression of SOD2 was detected in the single-cell datasets, which seems to be inconsistent with its mitigating effect previously confirmed [53, 54]. Therefore, the potential role of the gene is not determined simply by its expression. But regardless of whether the trend is consistent, abnormal expression of genes in multiple datasets requires more attention, which suggests the urgent need for further exploration of the underlying mechanism of these genes, especially with senescence. At the same time, other genes involved in our signature also deserve concern for the development of potential senolytics. INHBA conferred 5-FU chemoresistance mediated by cellular senescence in colon cancer cells through negative regulation of Hippo signaling [55]. Silencing of PAPSS2 can induce cellular senescence through augmented fibroblast growth factor receptor 1 (FGFR1) signaling [56, 57]. Moreover, up-regulation of BMP2 was reported to promote osteoblast differentiation and inhibit osteoblast senescence via increasing m6A methylation of Sp1 mRNA [58]. The multifunctional adhesion molecule CD44 was elevated in vascular endothelial cells to induce autophagy decline and aging phenotypes [59].

Although our current study has achieved some advanced results, there is still room for further exploration. Firstly, though the novel signature NP_Senescence exhibited excellent predictive efficacy in multiple external datasets, there is still a need for prospective cohorts to further validate its effectiveness. Secondly, we discovered the specific metabolic pattern in senescent NP cells. However, the regulatory mechanisms of some metabolic pathways, such as purine and pyrimidine synthesis, still need to be further excavated. Moreover, the roles of some senescence-related genes, like GLIS3, ANK3, and COL15A1, involved in the signature have not been validated in both in vitro and in vivo experiments, which are necessary and essential for an in-depth study and clinical application for IVDD patients in the future.

In summary, we shed light on exploring specific senescence characteristics in the degeneration of NP cells and intervertebral disc, and constructed a diagnostic model NP_Senescence based on the single cell algorithms hdWGCNA and scFEA. Meanwhile, NP cells in the high NP_Senescence score group exhibited the metabolic characteristic of improved synthesis in hyaluronic acid, pyrimidine, and purine. Finally, the novel signature was verified by the algorithm SenCID and validated in multiple external datasets, including two expression array datasets and one single cell dataset. These findings deepen the insights into the underlying genes involved in cellular senescence of NP cells and IVDD, providing potential therapeutic targets for IVDD patients.

## Supplementary Information

Below is the link to the electronic supplementary material.

ESM 1 (PNG 51.5 KB)

ESM 2 (XLSX 9.75 KB)

ESM 3 (XLSX 9.17 KB)

ESM 4 (XLSX 11.2 KB)

## Data Availability

The data in this study are available from the corresponding author upon reasonable request.
